# Supplementary figures and images for: USP5 enhances SGTA mediated protein quality control
Source: PLoS One. 2022 Jul 27;17(7):e0257786. doi: 10.1371/journal.pone.0257786 (PMC9328565; doi:10.1371/journal.pone.0257786)

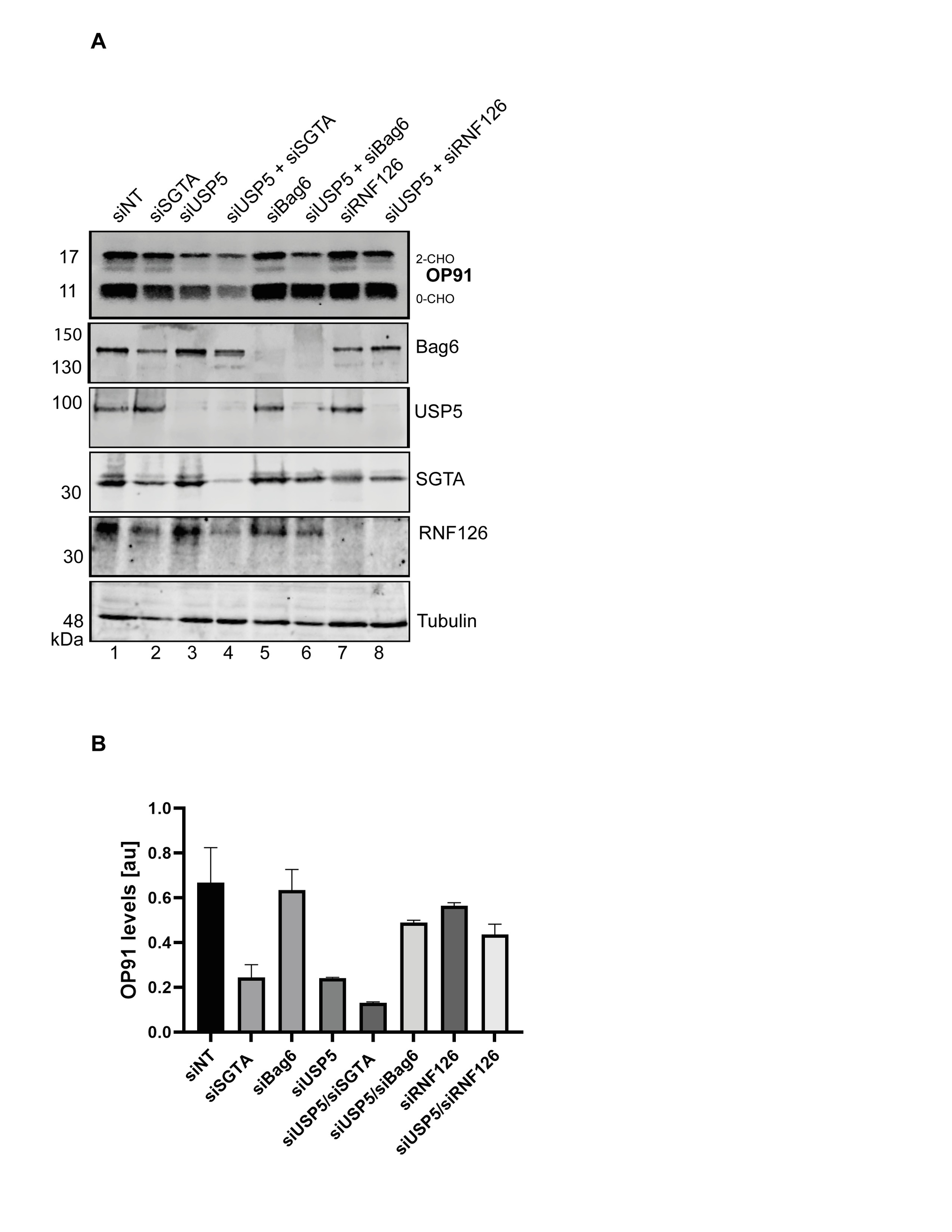

Supplement: S1 Fig — HeLa T-REx Flp-In cells stably expressing OP91 after induction were seeded at 50% confluence and transfected with 1 nM of siRNAi targeting either USP5, SGTA, BAG6 or RNF126 alone or in the combinations indicated. A nontargeting siRNAi was used as a negative control. After 48 hrs, OP91 expression was induced by replacing with medium containing 1 mg/ml of tetracycline. Cells were grown for 20 hrs post induction and total cell lysate were prepared and analysed by western blotting using antibodies for opsin (OP91), SGTA, USP5, BAG6 and tubulin (loading control) and detected using an Odyssey ® Fc imaging system. The siRNAi for RNF126, though not validated here was extensively used in previous studies [31]. (TIF) [file pone.0257786.s001.tif]

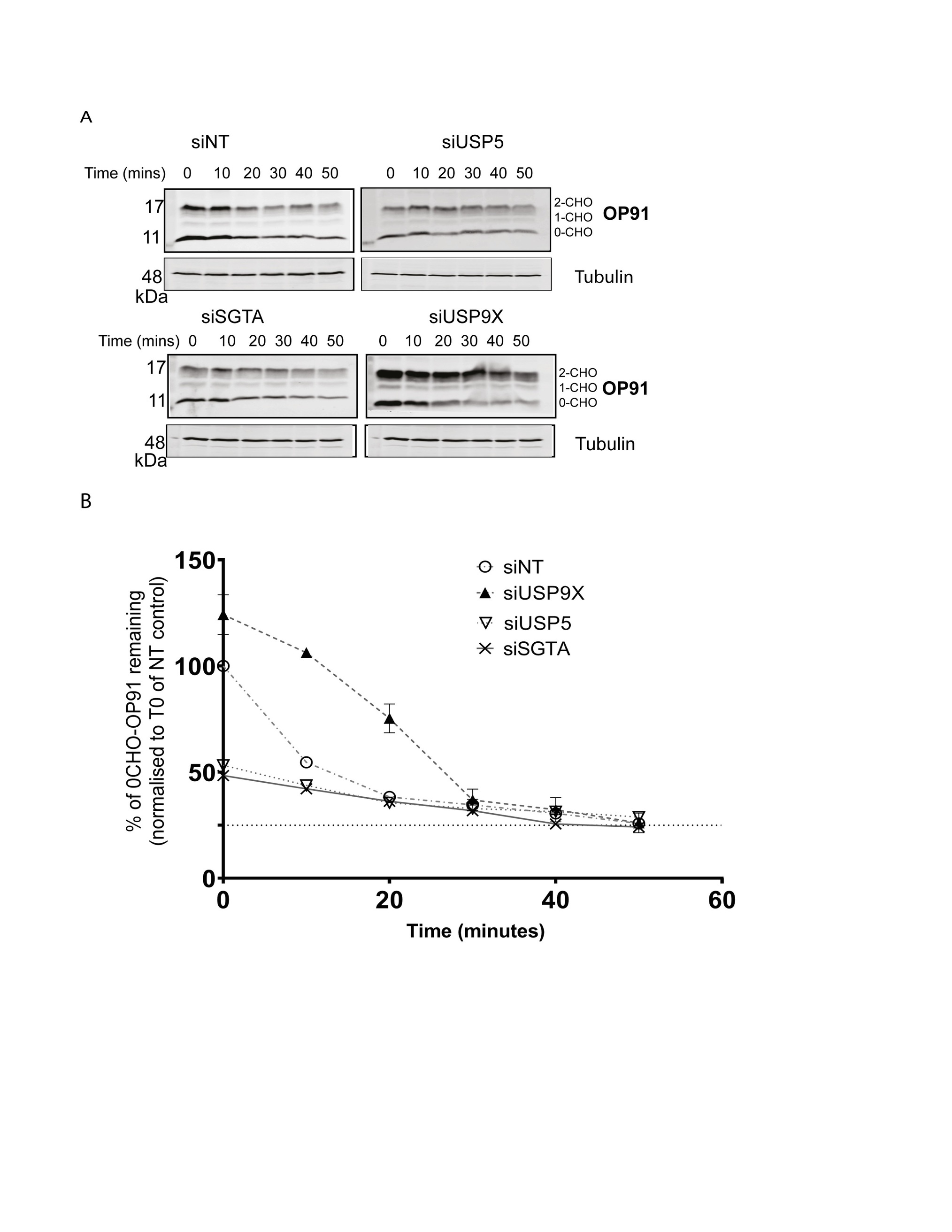

Supplement: S2 Fig — A) HeLa T-REx Flp-In cells stably expressing OP91 were seeded at 50% confluence and transfected with 1 nM of siRNAi targeting USP5, USP9X, SGTA or a nontargeting control and incubated for 48 hours. After this OP91 expression was induced by addition of medium containing 1 μg/ml of tetracycline and grown for 20 hrs post induction. Prior to harvesting transfected cells were treated with 100 μg/ml cycloheximide (Sigma, Aldrich) to inhibit protein synthesis. Cells were lysed directly into SDS-PAGE sample buffer at specific time-points followed by Western blotting with antibodies against opsin (OP91) and tubulin (loading control) using fluorescence- based detection (LICOR). B) OP91 signals were quantified using Odyssey ® Fc imaging system and normalised to the tubulin loading control, values show standard errors for n = 3. (TIF) [file pone.0257786.s002.tif]

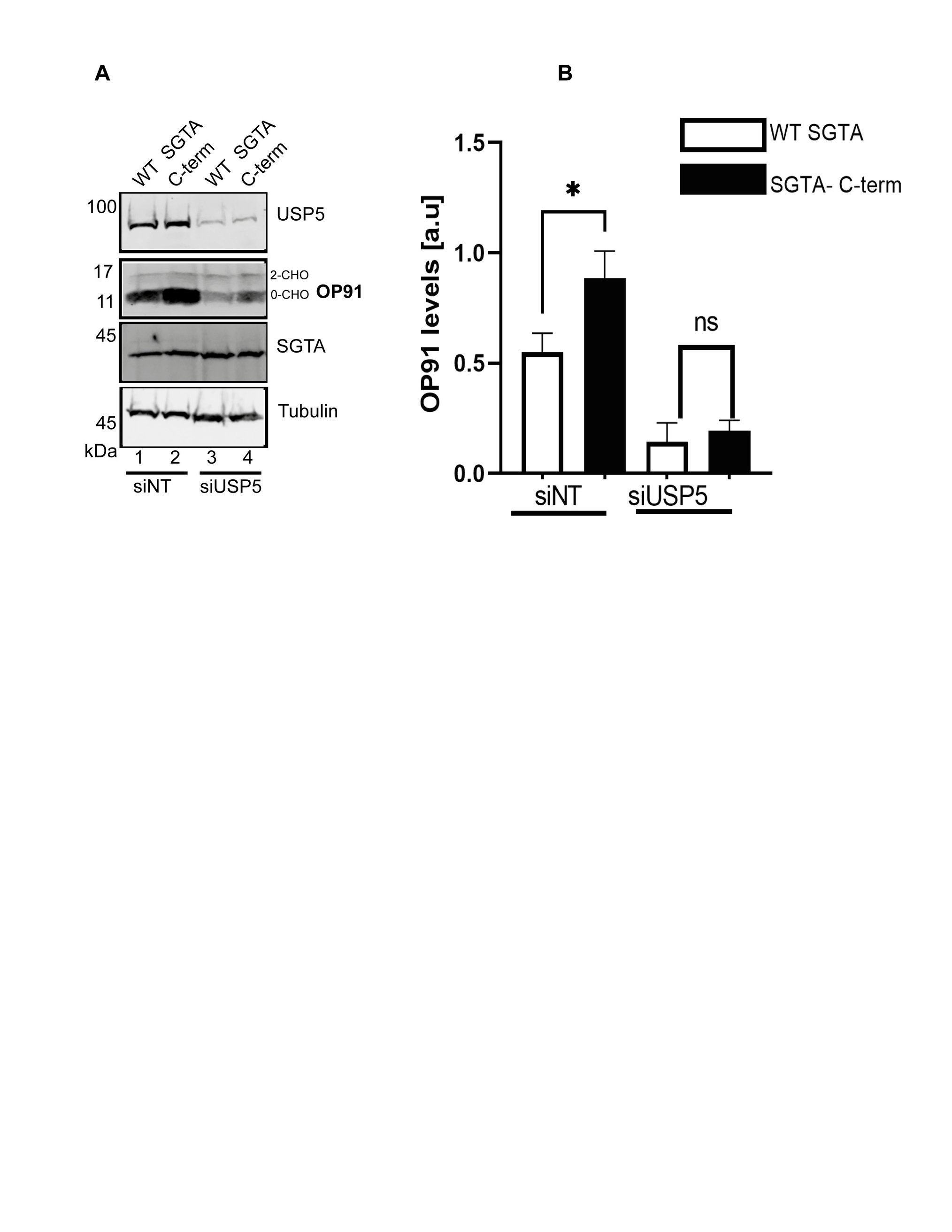

Supplement: S3 Fig — A) HeLa T-REx Flp-In cells stably expressing OP91 were seeded at 50% confluence and transfected with 1 nM of siRNAi targeting USP5 or a nontargeting control and incubated for 48 hours. Following this, cells were transfected with V5 tagged WT SGTA or SGTA-3xNNP/AAA C-terminal mutant (denoted here as SGTA C-term) followed by incubation for 6 hours before the medium was replaced with medium containing 1 mg/ml of tetracycline to induce OP91 expression. Cells were grown for 20 hrs post induction and total cell lysate were prepared and products analysed by western blotting using antibodies for opsin (OP91), V5 (SGTA and SGTA- C-term), and tubulin (loading control). B) OP91 signals were quantified using Odyssey ® Fc imaging system and normalised to the tubulin loading control, values show standard errors for n = 3. (TIF) [file pone.0257786.s003.tif]
