# Supplementary material for: USP5 enhances SGTA mediated protein quality control
Source: PLoS One. 2022 Jul 27;17(7):e0257786. doi: 10.1371/journal.pone.0257786 (PMC9328565; doi:10.1371/journal.pone.0257786)
Supplement: S1 Raw images — (PDF) [file pone.0257786.s004.pdf]

Figure 1A Results acquired by the Odyssey LI-COR imager

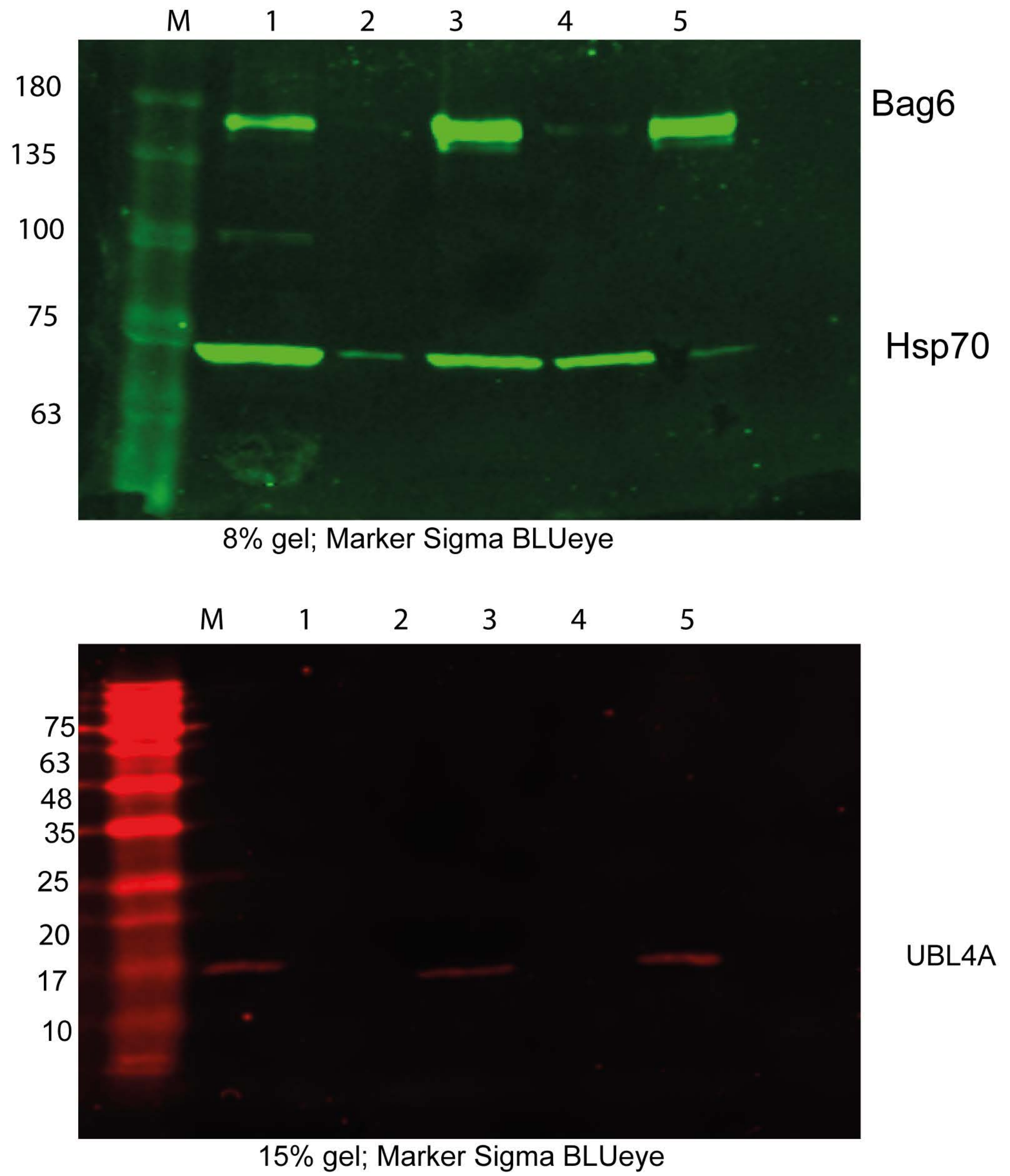

Figure 1B : all imades were acquired using the Odyssey LI-cor machine.

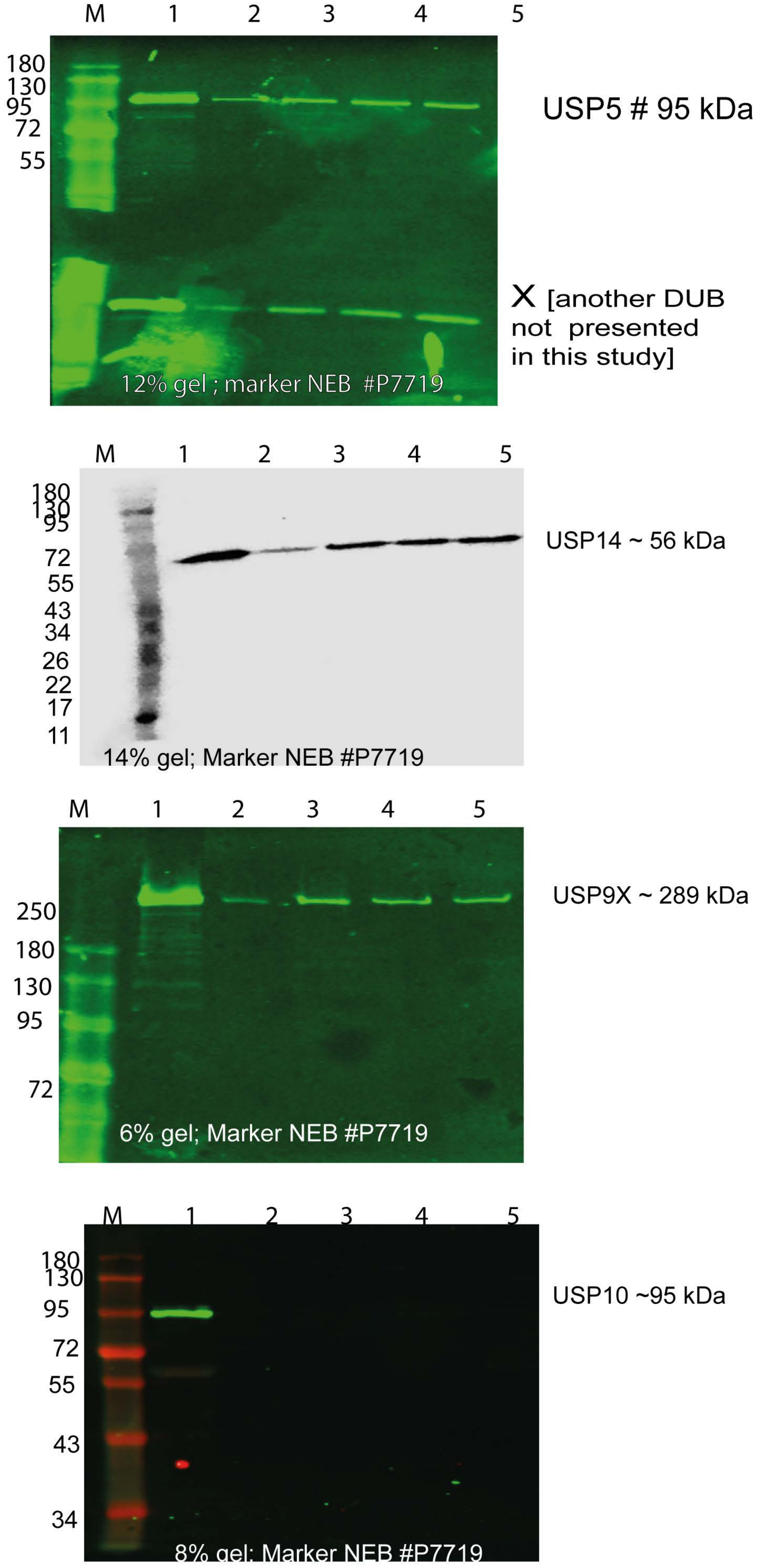

Figure 2A

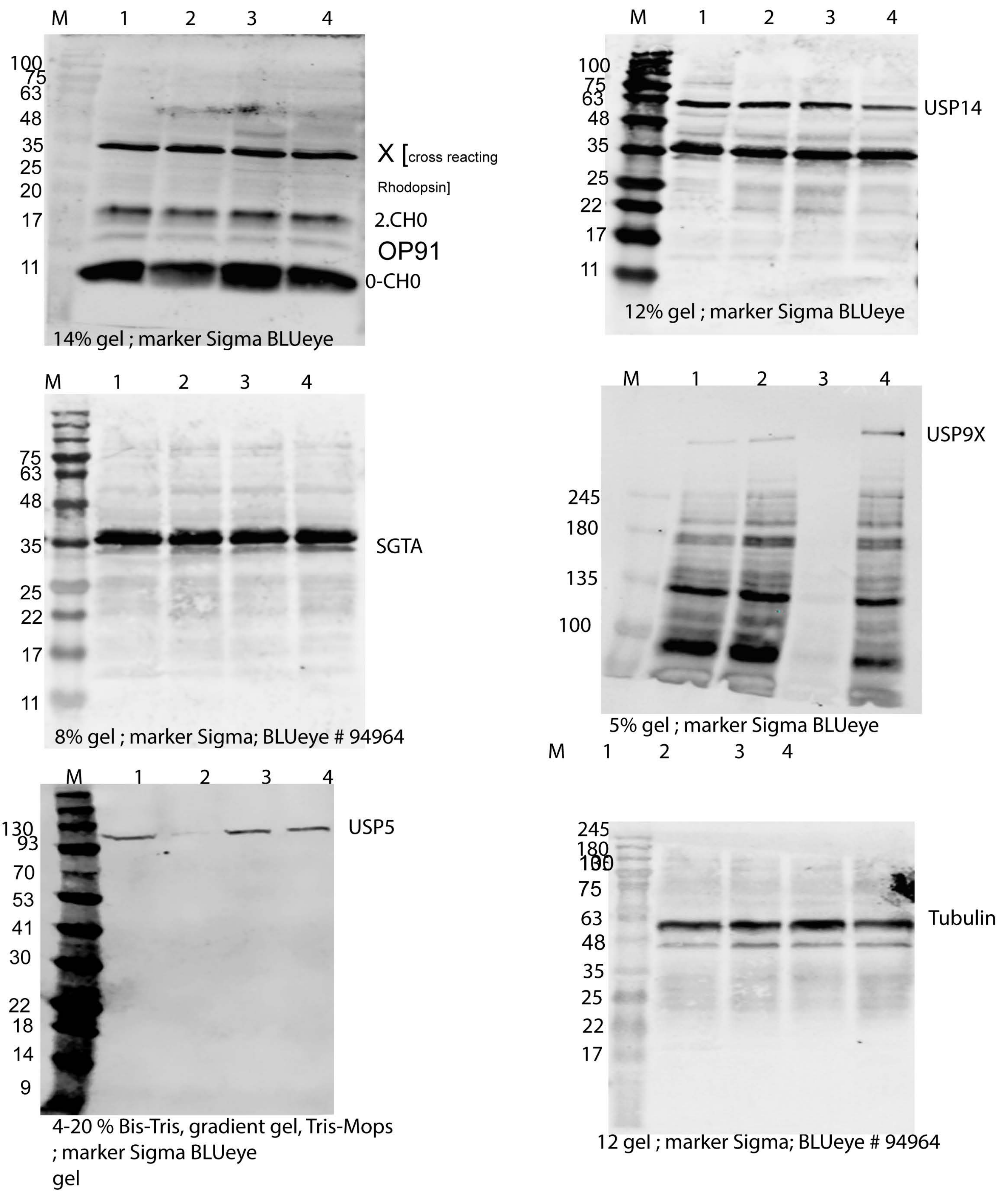

M 1 2 3 4
100
75
63
48
35
25
20
17
11
X [cross reacting Rhodopsin]
2.CH0
OP91
0-CH0
14% gel ; marker Sigma BLUeye
M 1 2 3 4
100
75
63
48
35
25
22
17
11
USP14
12% gel ; marker Sigma BLUeye
M 1 2 3 4
75
63
48
35
25
22
17
11
SGTA
8% gel ; marker Sigma; BLUeye # 94964
M 1 2 3 4
245
180
135
100
USP9X
5% gel ; marker Sigma BLUeye
M 1 2 3 4
130
93
70
53
41
30
22
18
14
9
USP5
4-20 % Bis-Tris, gradient gel, Tris-Mops
; marker Sigma BLUeye
gel
M 1 2 3 4
245
180
100
75
63
48
35
25
22
17
Tubulin
12 gel ; marker Sigma; BLUeye # 94964

Figure 2B: All images acquired using odyssey LI-COR

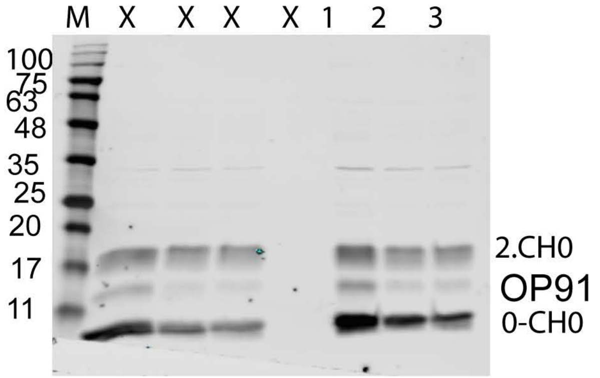

12 % gel ; marker : Sigma BLUeye

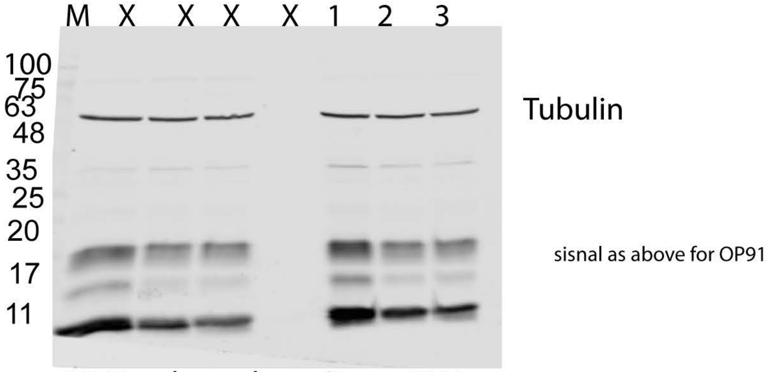

12 % gel ; marker : Sigma BLUeye

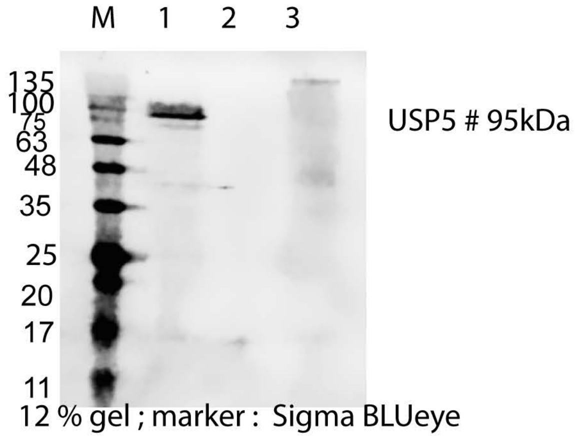

12 % gel ; marker : Sigma BLUeye

Figure 3: All images acquired using Odyssey LI-COR imaging

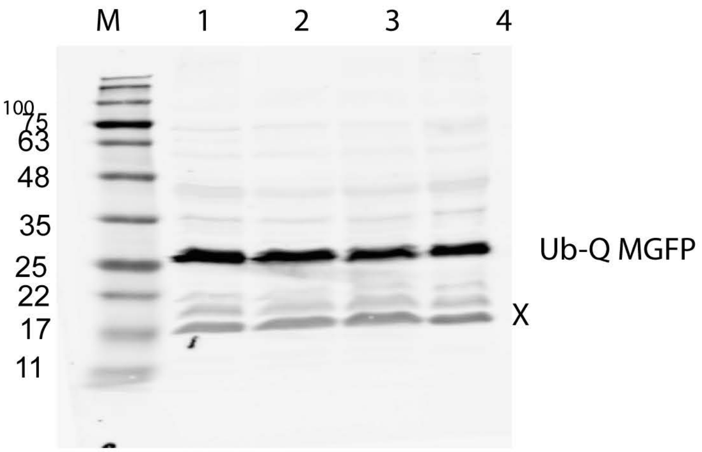

14% gel ; marker Sigma BLUeye

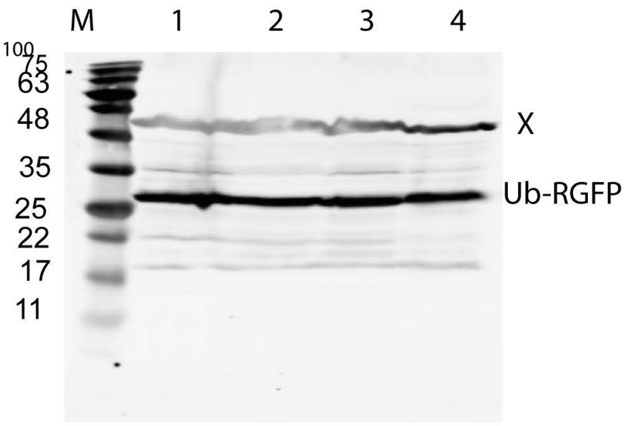

14% gel; Marker NEB #P7712

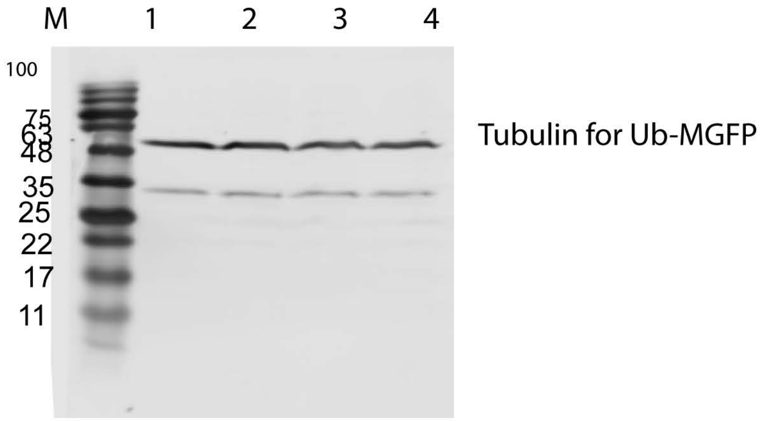

14% gel ; marker Sigma BLUeye

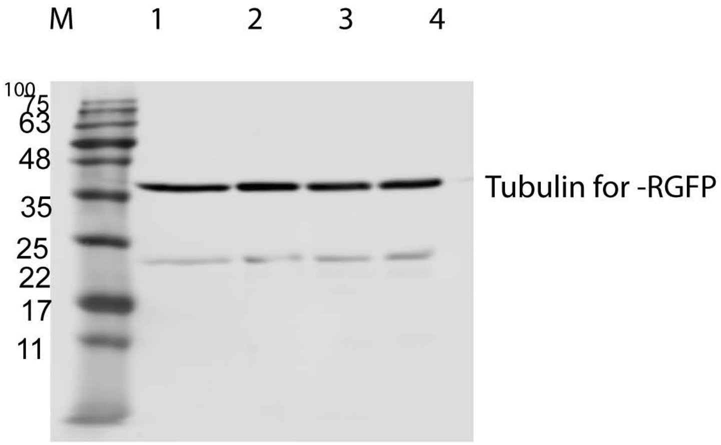

14% gel ; marker Sigma; BLUeye # 94964

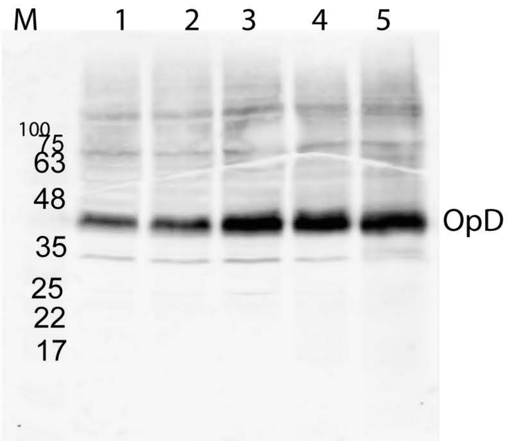

12% gel ; marker Sigma BLUeye

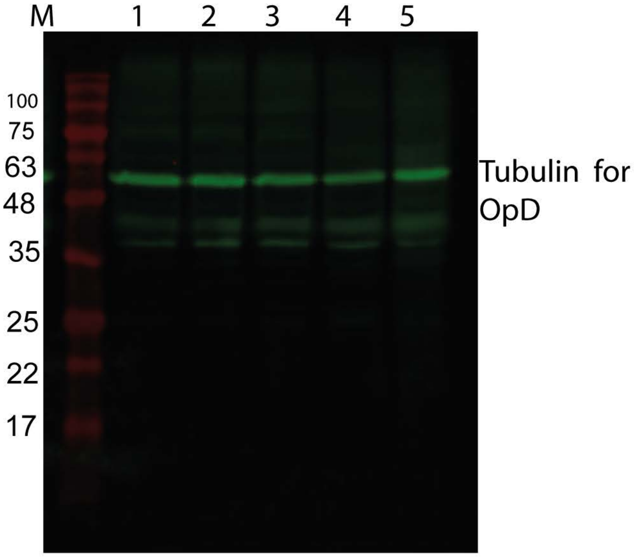

12% gel ; marker Sigma; BLUeye # 94964

Figure 4A: Acquired with LI-COR imaging machinre

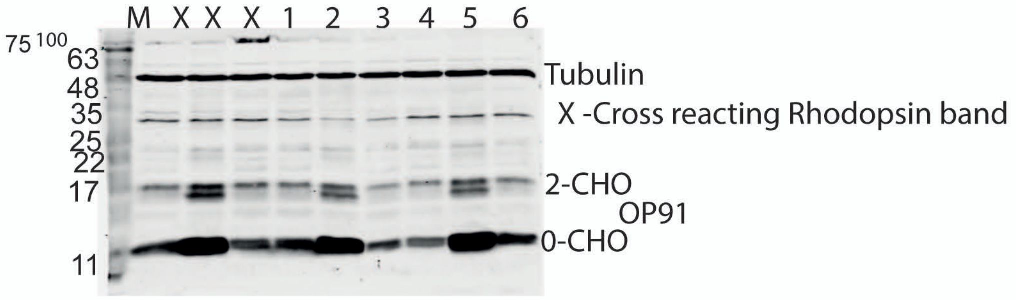

Figure 5A

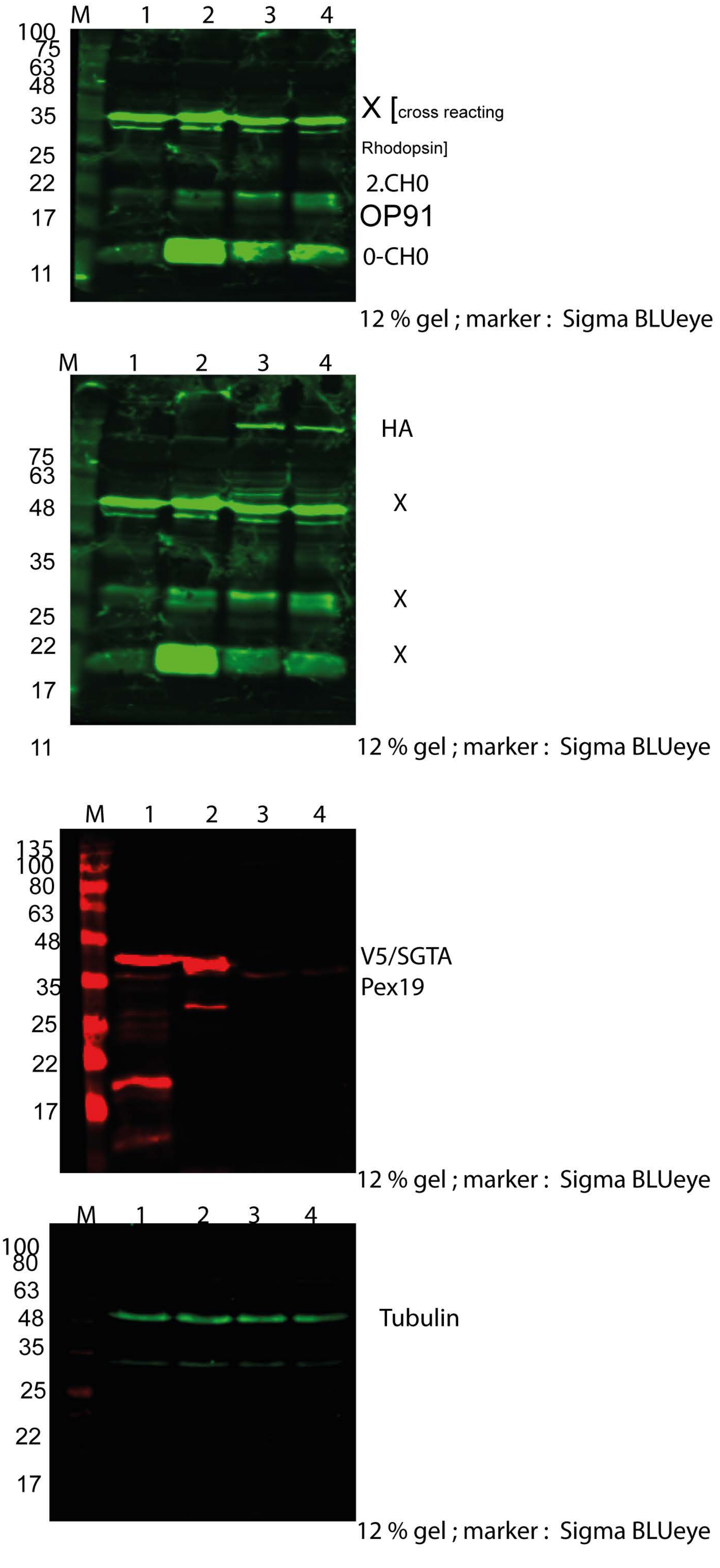

M 1 2 3 4
100
75
63
48
35
25
22
17
11
X [cross reacting
Rhodopsin]
2.CH0
OP91
0-CH0
12 % gel ; marker : Sigma BLUeye
M 1 2 3 4
HA
75
63
48
X
35
X
25
22
X
17
11
12 % gel ; marker : Sigma BLUeye
M 1 2 3 4
135
100
80
63
48
V5/SGTA
35
Pex19
25
22
17
12 % gel ; marker : Sigma BLUeye
M 1 2 3 4
100
80
63
48
Tubulin
35
25
22
17
12 % gel ; marker : Sigma BLUeye

Figure 5C. Cycloheximide chase, All figures were aquired using Odyssey LI-COR

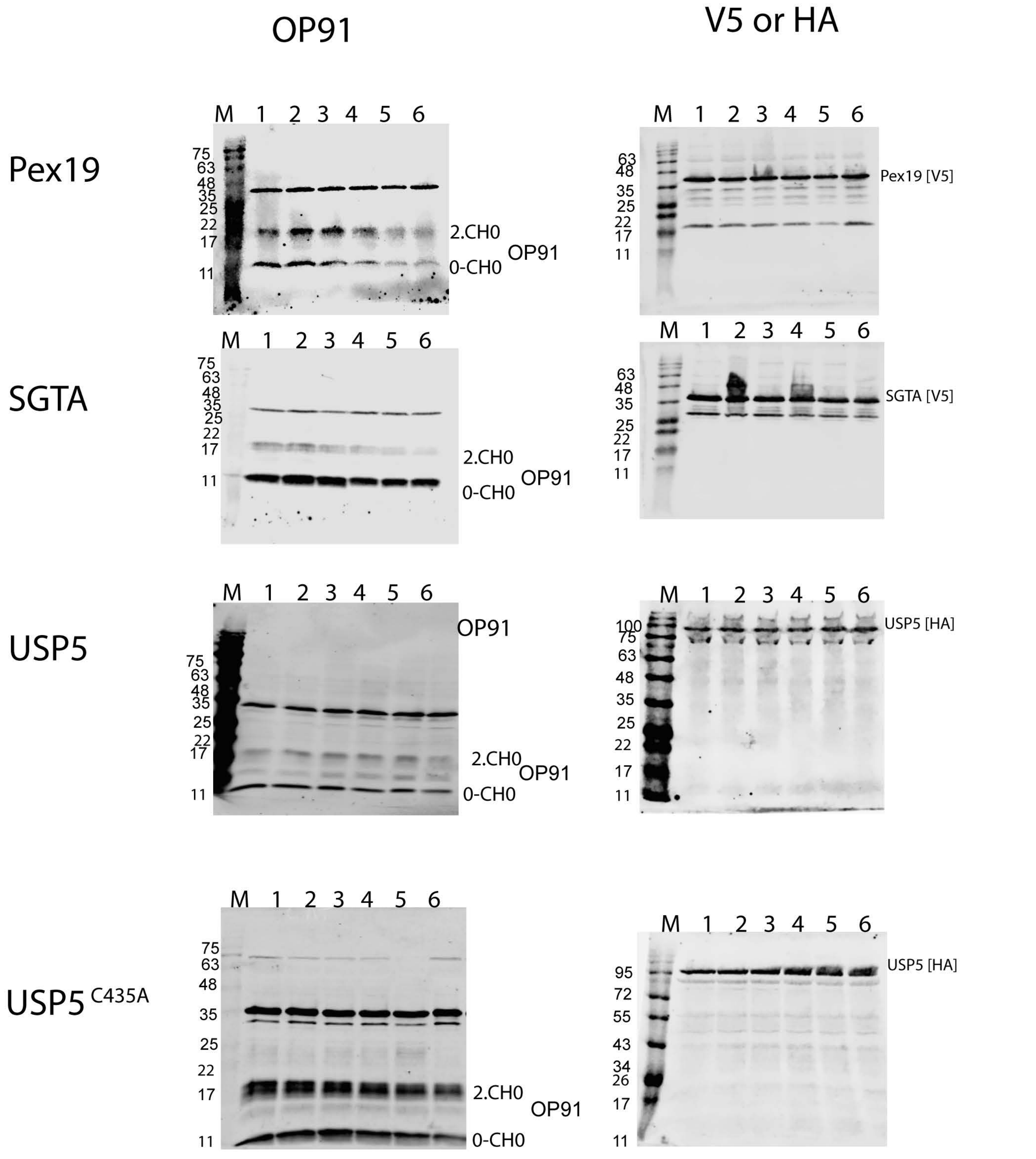

12 % gels ; marker : Sigma BLUeye marker for all except last gel on right with NEB #P7719

Figure 6A

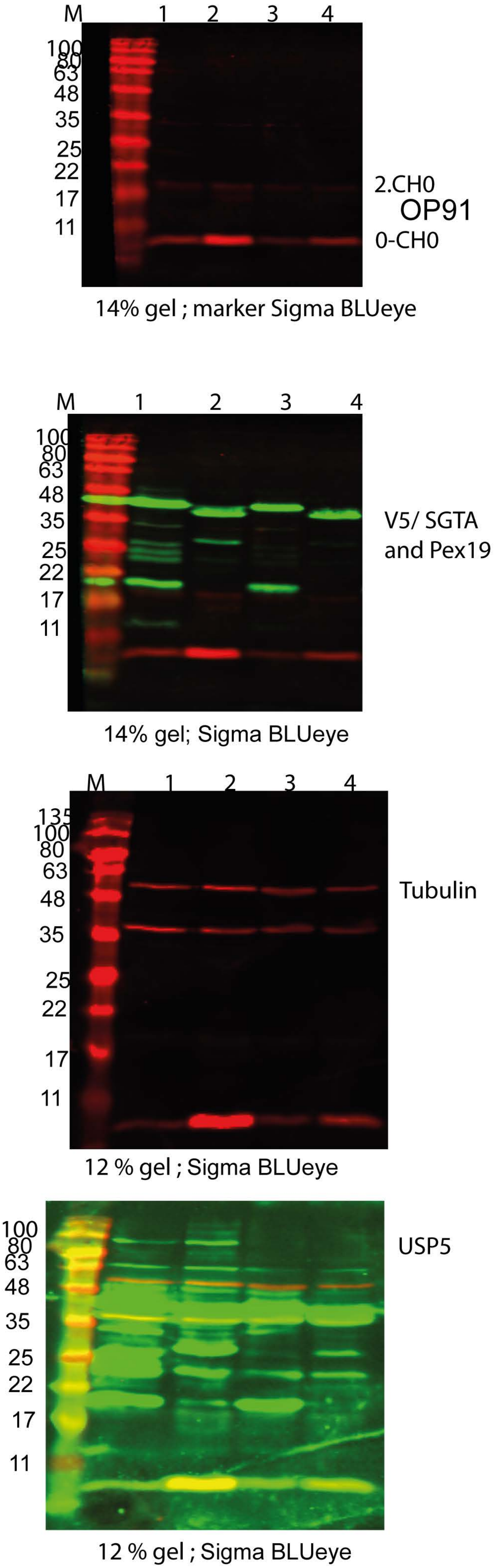

Figure 6B

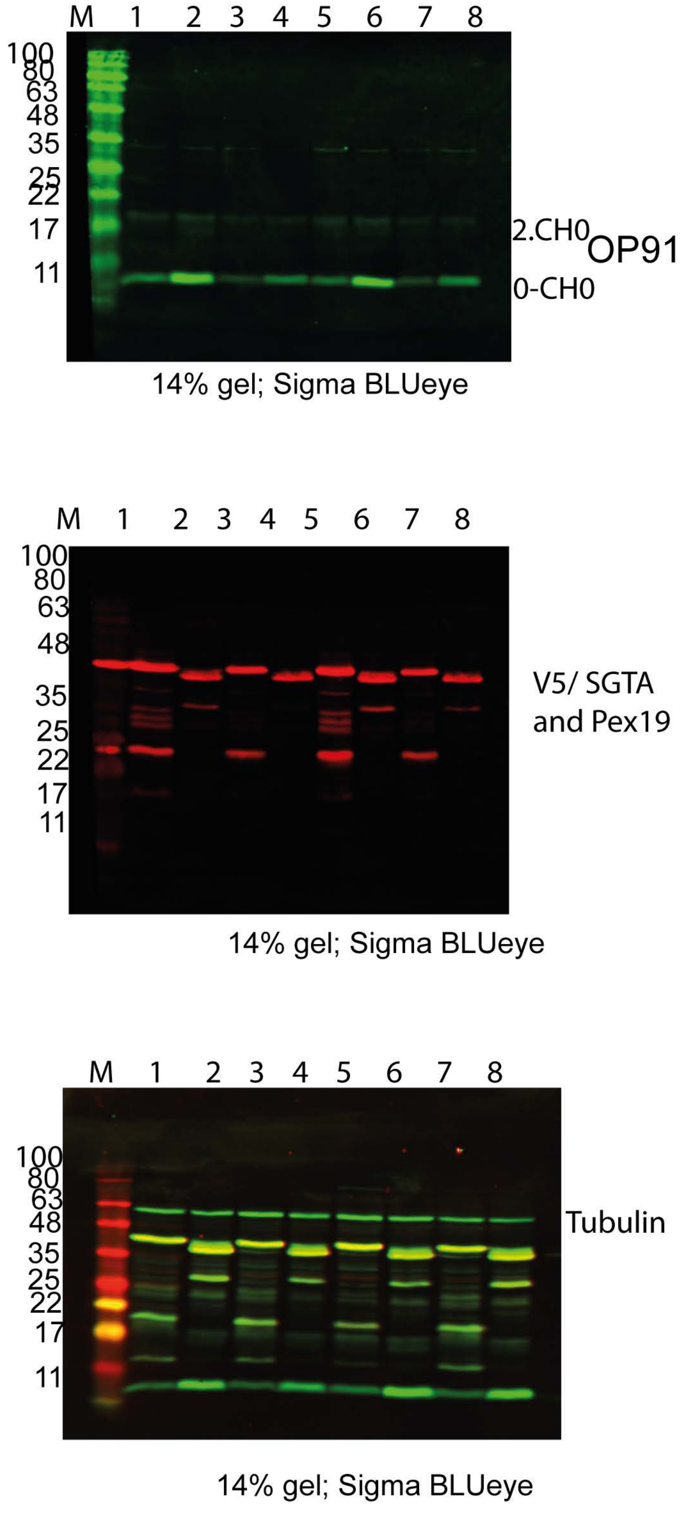

All images aqcuired using Odssey LI-COR machine

Figure 7A. Images aquired using LI-COR machine

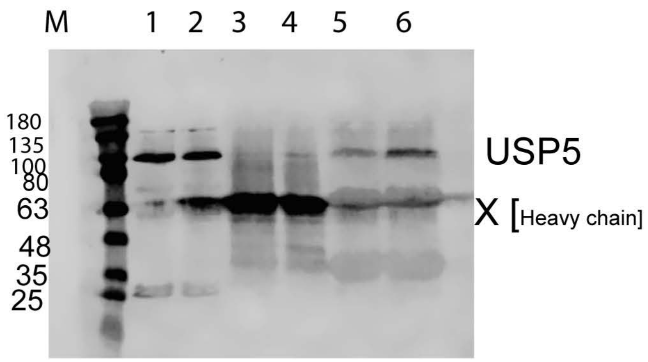

10 % gel ; Sigma BLUeye

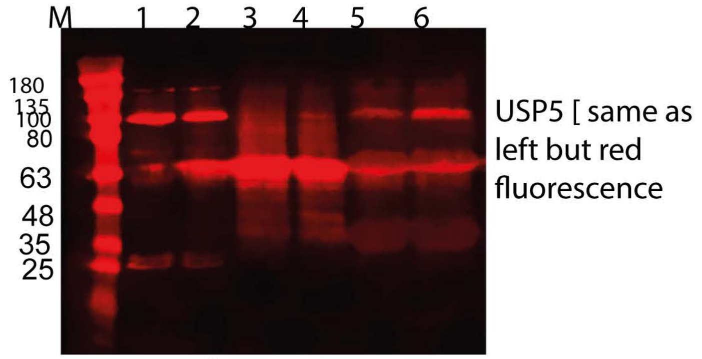

10 % gel ; Sigma BLUeye

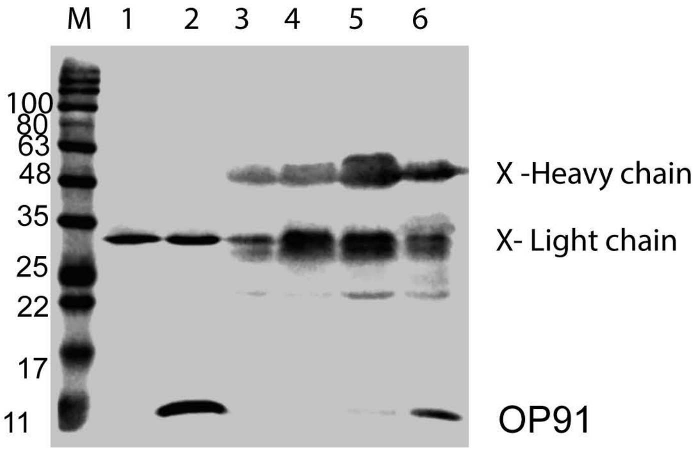

14 % gel ; Sigma BLUeye

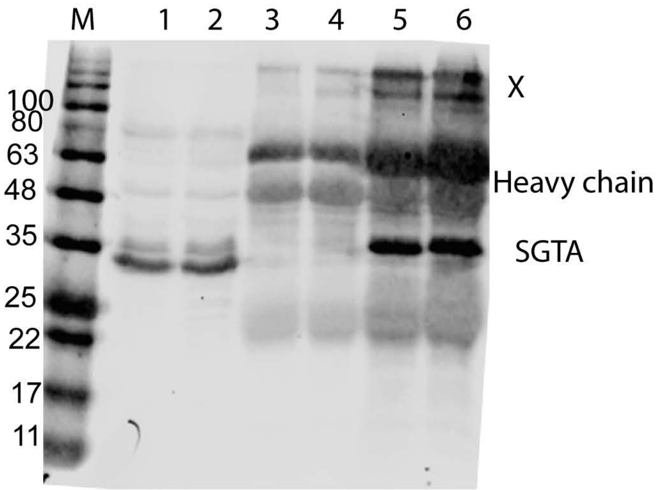

12 % gel ; Sigma BLUeye

Figure 7B: All images aqcuired using Odyssey LI-COR machine

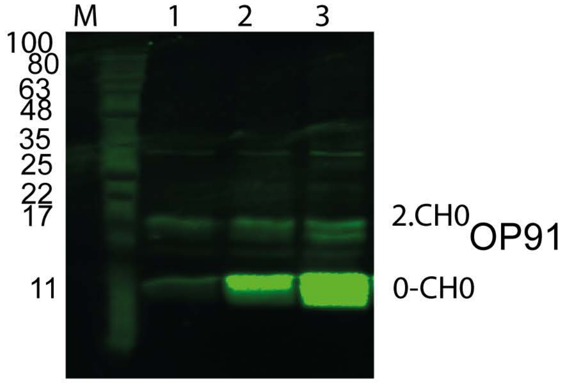

14 % gel ; Sigma BLUeye

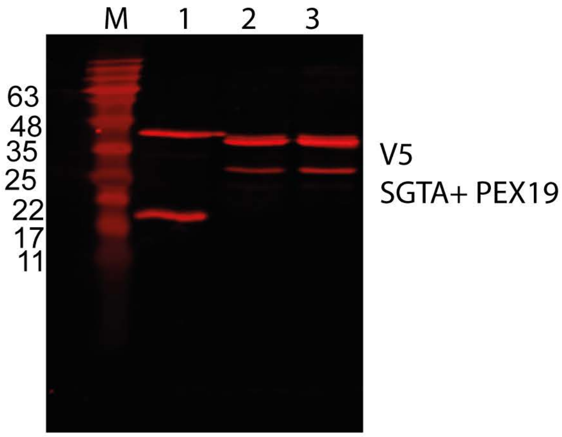

12 % gel ; Sigma BLUeye

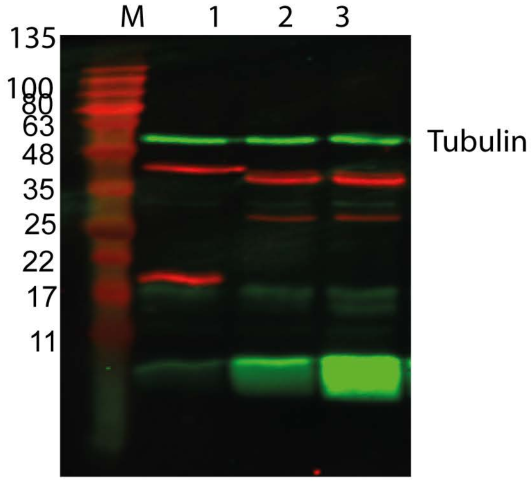

10 % gel ; Sigma BLUeye

Figure 7C

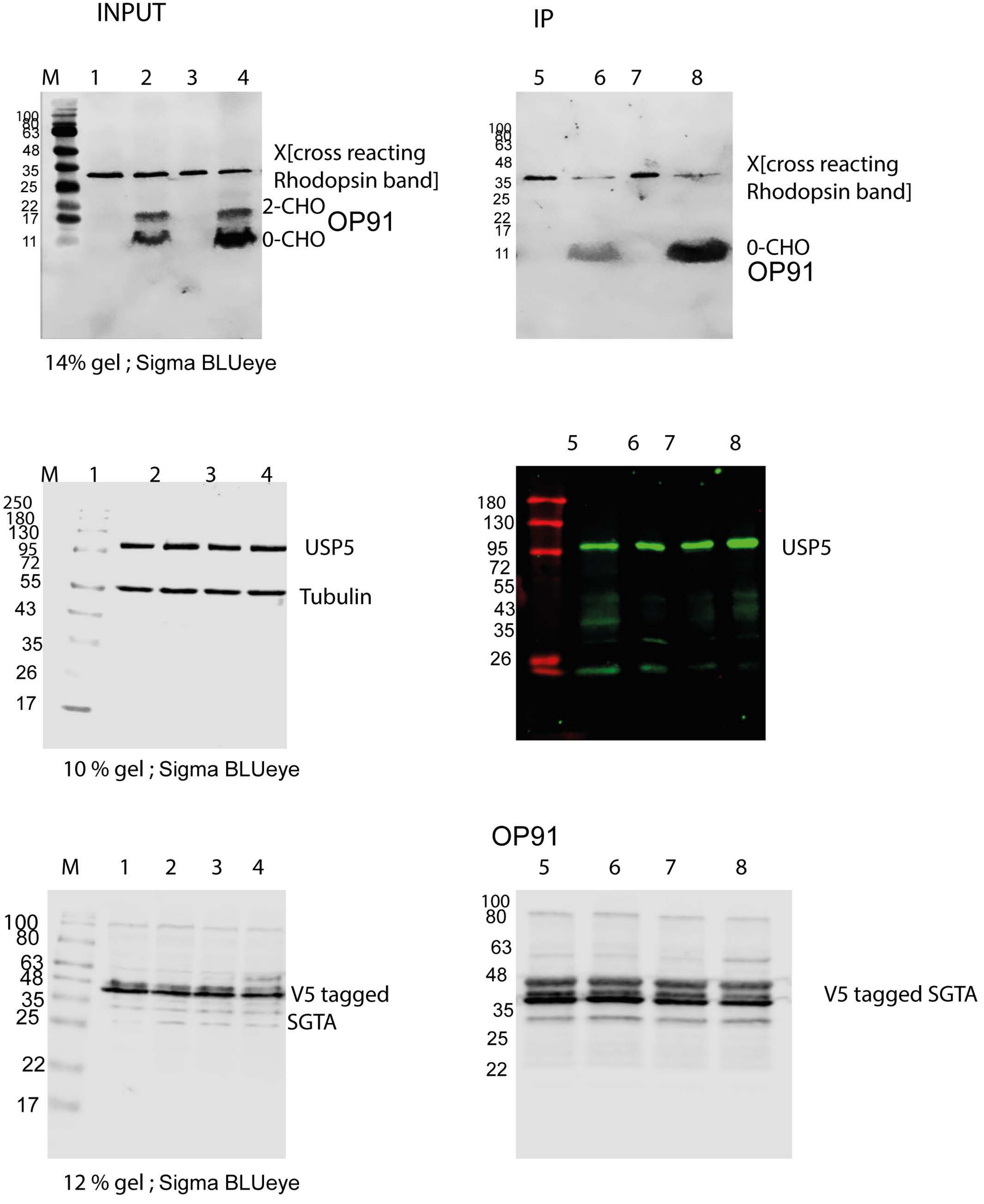
INPUT
IP
M 1 2 3 4
5 6 7 8
100
80
63
48
35
25
22
17
11
X[cross reacting
Rhodopsin band]
2-CHO
0-CHO
OP91
14% gel ; Sigma BLUeye
250
180
130
95
72
55
43
35
26
17
USP5
Tubulin
10 % gel ; Sigma BLUeye
OP91
V5 tagged
SGTA
V5 tagged SGTA
12 % gel ; Sigma BLUeye

Figure 7E

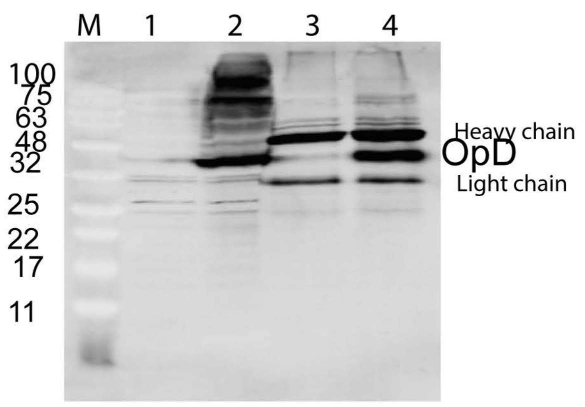

14 % gel ; Sigma BLUeye

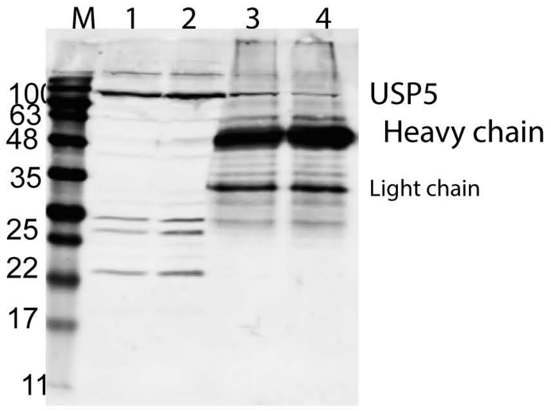

14 % gel ; Sigma BLUeye

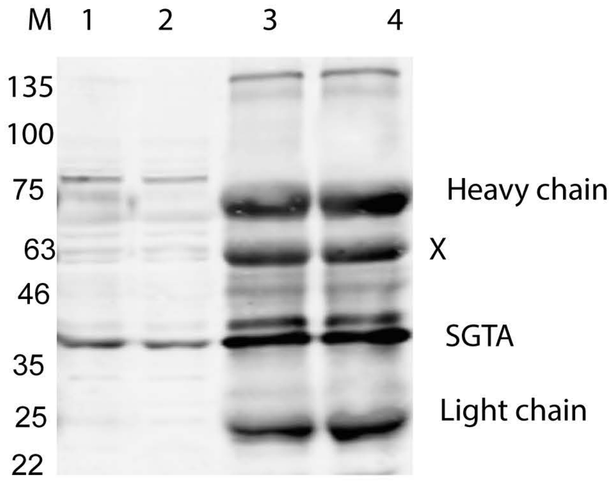

12 % gel ; Sigma BLUeye

S1A Fig.

x = Cross reacting band

All blots captured using Near IR fluorescence on an LICOR Odyssey XF

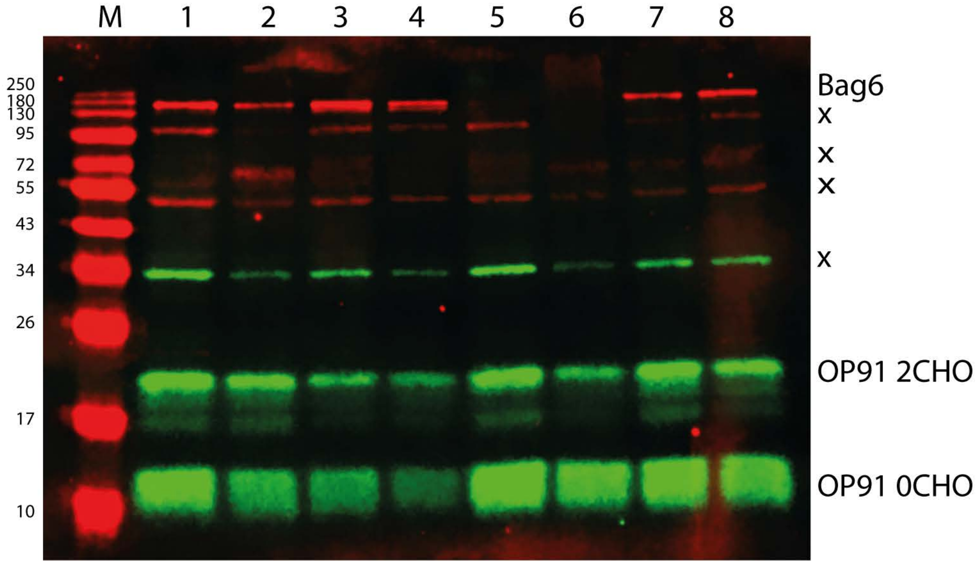

14% gel; Marker NEB #P7719

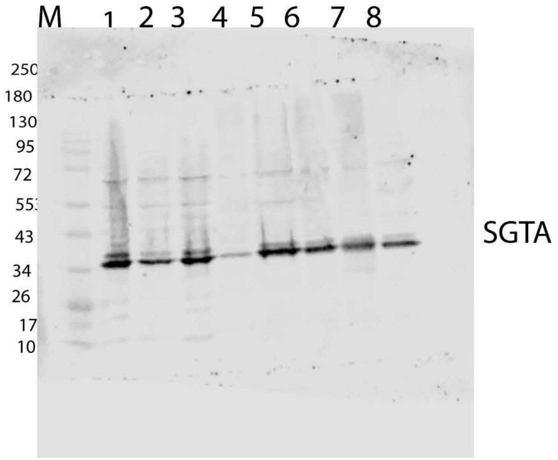

14% gel; Marker NEB #P7719

S1A Fig.

x = Cross reacting band

All blots captured using Near IR fluorescence on an LICOR Odyssey XF

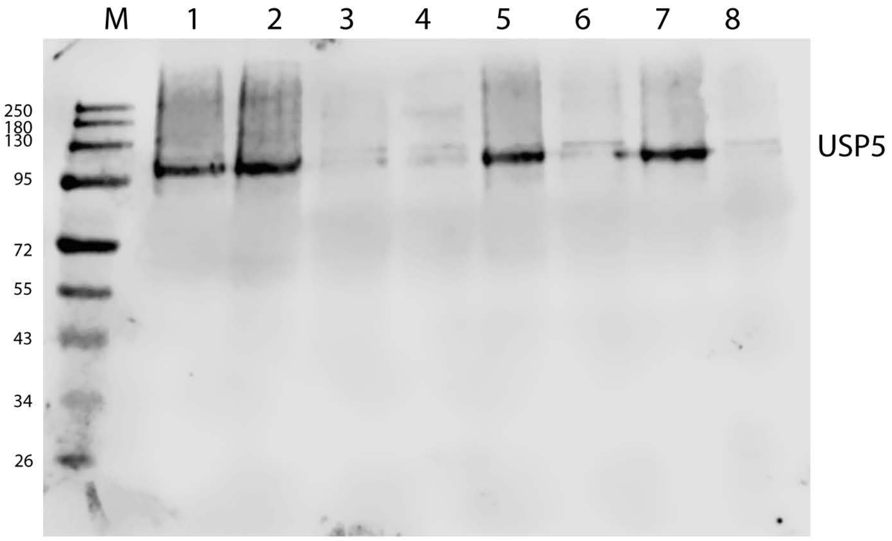

12% gel; Marker NEB #P7719

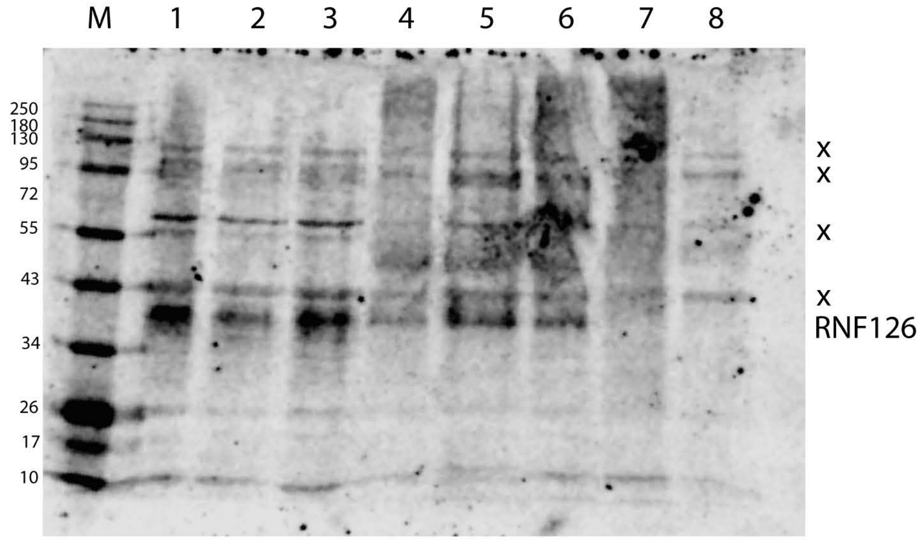

14% gel; Marker NEB #P7719

Sup Fig 2A

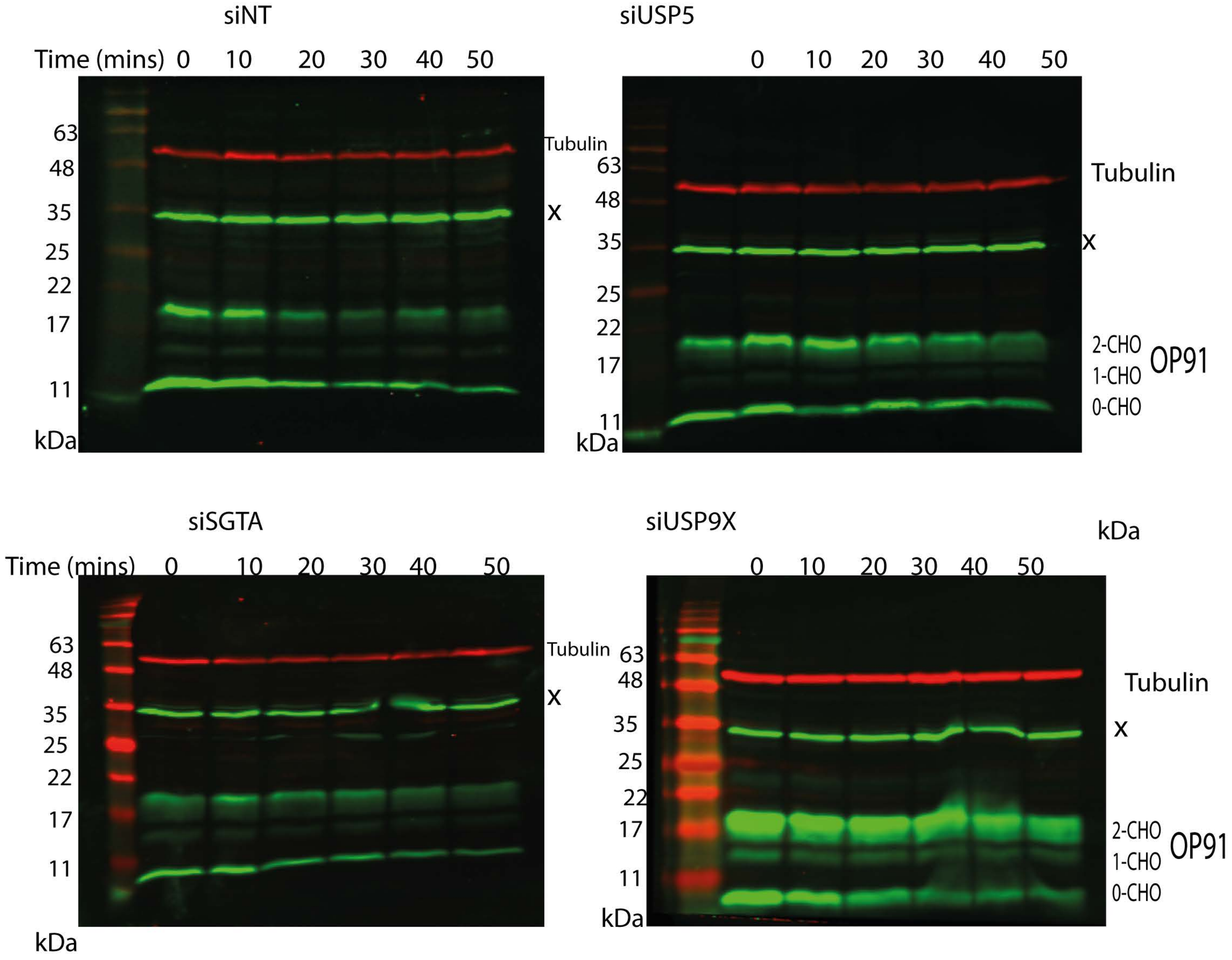
siNT
Time (mins) 0 10 20 30 40 50
63
48
35
25
22
17
11
kDa
Tubulin
x
siUSP5
0 10 20 30 40 50
63
48
35
25
22
17
11
kDa
Tubulin
x
2-CHO
1-CHO
0-CHO
OP91
siSGTA
Time (mins) 0 10 20 30 40 50
63
48
35
25
22
17
11
kDa
Tubulin
x
siUSP9X
kDa
0 10 20 30 40 50
63
48
35
25
22
17
11
kDa
Tubulin
x
2-CHO
1-CHO
0-CHO
OP91

S3A Fig.

x = Cross reacting band

All blots captured using Near IR fluorescence on an LICOR Odyssey XF

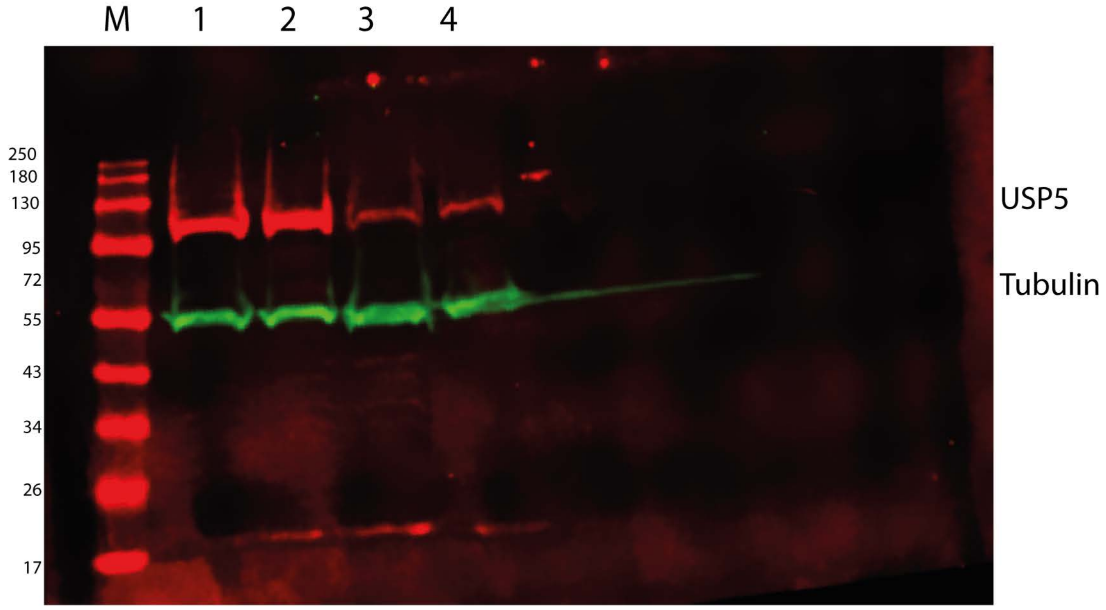

10% gel; Marker NEB #P7719

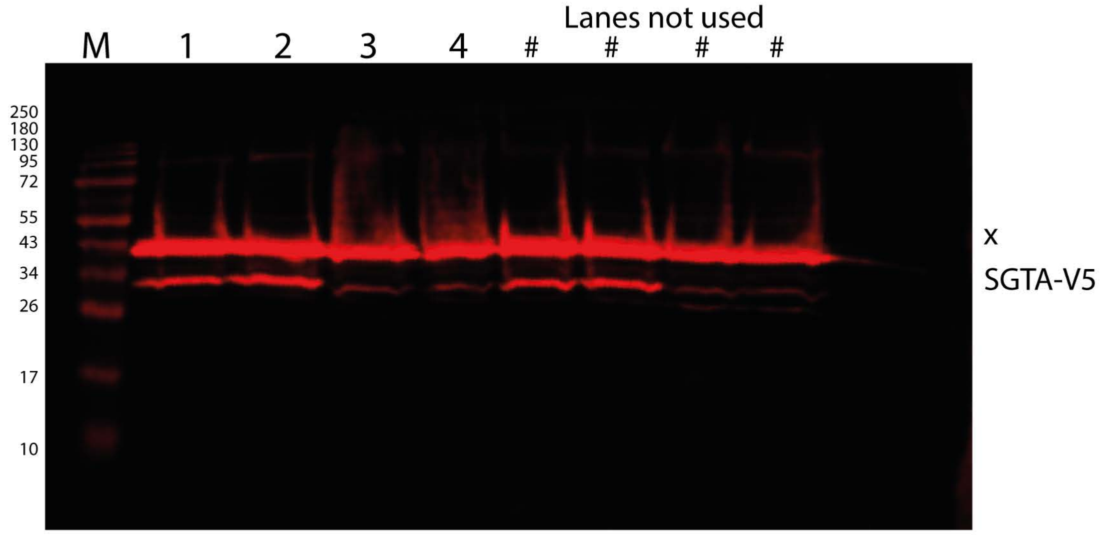

14% gel; Marker NEB #P7719

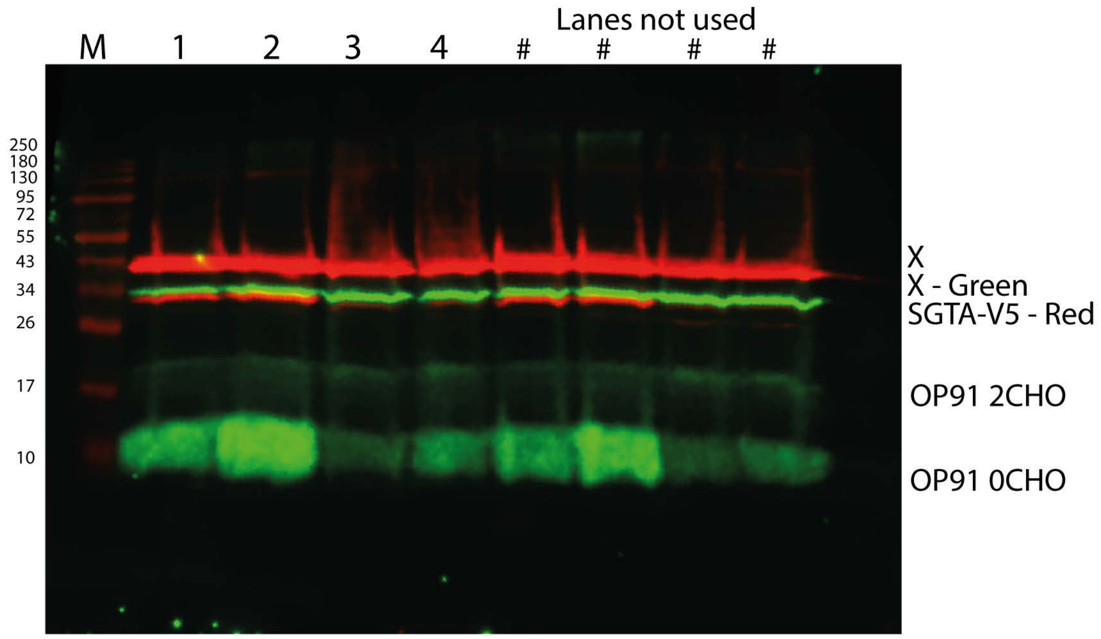

14% gel; Marker NEB #P7719
